# Beyond Clinical Acuity: Cardiac Comorbidity and Complication Profiles and the Prehospital Hospitalization/Transport Decision in Ischemic Heart Disease—A Five-Year Retrospective Emergency Medical Service Study in Astana, Kazakhstan

**DOI:** 10.3390/healthcare14152308

**Published:** 2026-07-31

**Authors:** Akerke Chayakova, Oxana Tsigengagel, Gulzira Zhussupova

**Affiliations:** 1 Department of Epidemiology and Biostatistics, Astana Medical University, Astana 010000, Kazakhstan; chayakova.a@amu.kz; 2 “SANAT” National Education Development Science Center, Astana 010000, Kazakhstan; gulzira1970@gmail.com

**Keywords:** ischemic heart disease, emergency medical services, hospitalization, comorbidity, heart failure, cardiogenic shock, logistic regression, prehospital care, Kazakhstan

## Abstract

**Highlights:**

**What are the main findings?**

- In a five-year call-level cohort of 9985 EMS activations for ischemic heart disease in Astana, the study quantified the real prehospital disposition decision: 26.8% of calls resulted in hospitalization/transport and 73.2% were managed without hospital transfer.
- The expected high-acuity predictors—cardiogenic shock and acute/unstable IHD—dominated the decision, while the novel contribution is the independent quantification of routinely coded cardiac comorbidity profiles, especially heart failure, other arrhythmias and atrial fibrillation, in an understudied Central Asian EMS system.

**What are the implications of the main findings?**

- Routinely recorded comorbidity and complication fields should be interpreted as candidate inputs for future, prospectively validated field risk-stratification models, not as a ready-to-use decision tool.
- At the system level, aggregated comorbidity-aware patterns may help design future audits of dispatch, crew allocation and non-conveyance safety, provided that richer clinical data and outcome linkage are added.

**Abstract:**

**Background/Objectives:** Ischemic heart disease (IHD) is a major cause of cardiovascular mortality, and emergency medical service (EMS) crews often make the first decision on whether an IHD-coded patient should be transported to hospital or managed at the scene. Evidence from Central Asian EMS systems is sparse, and it remains unclear whether routinely coded comorbidity information adds decision-relevant information beyond acute presentation. We aimed to identify predictors of the EMS hospitalization/transport decision among IHD calls in Astana, Kazakhstan. **Materials and Methods:** We conducted a retrospective call-level cohort study of 9985 consecutive EMS calls coded as IHD (ICD-10 I20-I25) over a five-year period. The endpoint was field disposition—hospitalization/transport versus being left at the scene—and should not be interpreted as confirmed ACS, mortality, clinical appropriateness or any other patient outcome. Group comparisons used the Mann–Whitney U and Pearson chi-square tests, and independent associations were estimated using explanatory multivariable logistic regression. **Results:** Overall, 2676 calls (26.8%) resulted in hospitalization/transport. The strongest independent predictors were cardiogenic shock (aOR 15.06), acute/unstable IHD versus chronic I25 (aOR 8.52) and heart failure (aOR 2.46). Other arrhythmias (aOR 1.84), atrial fibrillation (aOR 1.60), male sex (aOR 1.65) and age <45 years (aOR 1.88) were also associated with higher transport odds, whereas age ≥75 years (aOR 0.61), specialized crews (aOR 0.84) and high dispatch urgency (categories 1–2; aOR 0.84) were associated with lower odds. Model discrimination was moderate (AUC 0.69; optimism-corrected AUC 0.69), plausibly reflecting the absence of ECG findings, vital signs, symptom severity and hospital outcome data. **Conclusions:** The expected acuity markers dominated EMS transport decisions, but routinely coded cardiac comorbidities were independently associated with disposition in this understudied setting. The model is not deployable for individual triage; these variables should be considered candidate inputs for future models that incorporate richer clinical data, outcome linkage and prospective validation.

## 1. Introduction

Ischemic heart disease (IHD) remains the leading cardiovascular cause of death worldwide, and its burden is increasing as populations age and cardiometabolic risk accumulates [1,2,3,4]. The burden is especially relevant for low- and middle-income or transitioning health systems, where registry evidence remains comparatively limited [4,5]. In Kazakhstan and the wider Central Asian region, cardiovascular disease dominates the mortality profile while risk-factor control remains incomplete [6]. In this setting, the emergency medical service (EMS) is often the first clinical contact for patients with cardiac symptoms, and crews must decide at the scene whether an IHD-coded patient requires hospital transport or can be managed without transfer.

Most prehospital coronary research has focused on acute coronary syndrome, treatment delay, PCI access, mortality or downstream adverse outcomes rather than on the operational conveyance decision itself [7,8,9,10]. A newer triage literature has shown that registry, dispatch, electrocardiographic and machine learning models can support risk stratification for chest pain or non-conveyance, usually reporting moderate discrimination and emphasizing the safety risks of both over-triage and under-triage [10,11,12,13,14,15,16]. These studies provide the methodological basis for using routinely collected EMS data, but they do not fully answer how crews make the binary at-scene decision to transport or not transport patients with IHD in routine urban practice.

Comorbidity may be particularly important in routine IHD care because many EMS activations involve chronic coronary disease rather than time-critical STEMI/NSTEMI. Heart failure, atrial fibrillation, other arrhythmias and cardiogenic shock are strongly associated with emergency admission, adverse outcomes or escalation of care in hospital-based and registry studies [17,18,19,20,21,22,23,24,25,26,27,28], and demographic effects on ACS presentation and treatment are well documented [29,30,31]. However, it remains uncertain whether the comorbidity and complication profile recorded by ambulance crews independently shapes the prehospital hospitalization/transport decision after adjustment for acute clinical form, demographic factors and operational variables. This uncertainty is especially important in Central Asian urban EMS systems, where local evidence is scarce and externally derived risk tools may not transfer directly.

To address this gap, we analyzed five years of EMS calls for IHD in Astana, Kazakhstan. The novelty of the study lies not in showing that shock or acute IHD prompt transport—which is clinically expected—but in quantifying, at the call level, whether routinely coded cardiac comorbidities and complications add independent information to a real EMS disposition decision in an understudied setting. The objectives were: (i) to describe demographic, clinical, comorbidity and operational characteristics by EMS disposition; (ii) to identify independent predictors of hospitalization/transport using multivariable logistic regression; and (iii) to estimate the relative contribution of comorbidity and complication fields compared with acuity, sex, age and operational factors.

## 2. Materials and Methods

### 2.1. Study Design and Setting

We performed a retrospective, observational cohort study of emergency medical service (EMS) calls attended for ischemic heart disease in Astana, the capital of Kazakhstan, over an approximately five-year period from 19 February 2020 to 30 June 2024. The final year (2024) therefore covers the first half of the calendar year only, which should be borne in mind when interpreting annual call volumes. Astana is served by a centralized municipal ambulance dispatch system that records each call electronically, including dispatch priority, crew type, response and on-scene time intervals, working diagnosis (coded to ICD-10) and final disposition. The study followed the principles of the Declaration of Helsinki, and only de-identified routinely collected operational data were analyzed. Reporting adheres to the STROBE recommendations for observational studies.

### 2.2. Participants and Case Definition

**Figure 1:**
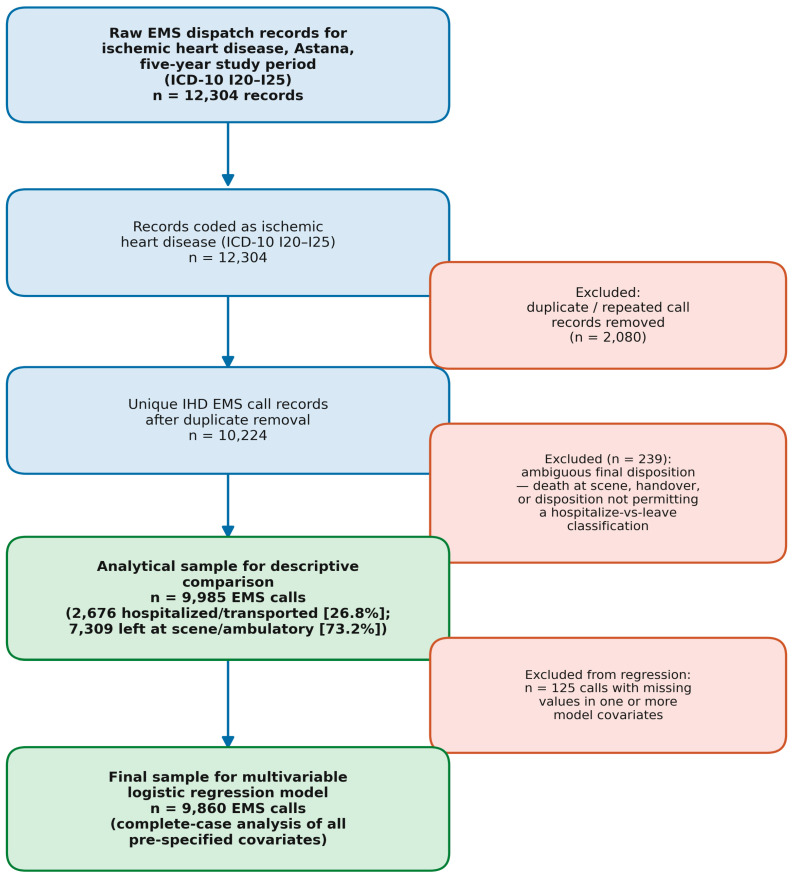
Data-processing and cleaning flow from the raw EMS dispatch dataset to the final analytical samples. Starting from 12,304 raw EMS dispatch records coded as ischemic heart disease (ICD-10 I20–I25) over the five-year study period, 2080 duplicate call records were removed, leaving 10,224 unique call records. A further 239 calls with an ambiguous final disposition (death at scene, handover, or any disposition that did not permit a hospitalize-versus-leave classification) were excluded, yielding the descriptive analytical sample of 9985 calls (2676 hospitalized/transported [26.8%] and 7309 left at scene/ambulatory [73.2%]). Finally, 125 calls with missing values in one or more pre-specified model covariates were excluded to give the complete-case sample of 9860 calls used in the multivariable logistic regression model.

Eligible records were all EMS calls with a working diagnosis of ischemic heart disease, defined by ICD-10 codes I20–I25. In keeping with registry-based AMI studies that identify cases through principal discharge or working diagnoses [9], calls were classified into two clinical forms: acute/unstable IHD (unstable angina and acute coronary syndromes, including I20–I24) and chronic IHD (I25). Comorbidities and complications documented by the crew were captured as binary indicators using their respective ICD-10 codes: diabetes mellitus (E10–E14), chronic obstructive pulmonary disease and related obstructive disease (J40–J47), arterial hypertension (I10–I15), heart failure (I50), atrial fibrillation (I48), other arrhythmias (I47, I49), cardiogenic shock (R57.0) and pneumonia (J12–J18). After removal of 2080 duplicate call records from the 12,304 raw IHD-coded dispatch records and exclusion of 239 records with a final disposition of death at scene or handover that did not permit a hospitalize-versus-leave classification, 9985 calls were available for descriptive comparison and 9860 for the fully adjusted regression model. The complete data-processing and cleaning cascade is summarized in Figure 1. The unit of analysis was the individual EMS call rather than the unique patient: each dispatch record represents one ambulance activation and the conveyance decision taken at that encounter. Because the dispatch dataset did not contain a stable patient identifier that could be reliably linked across calls, repeat calls by the same patient could not be collapsed to the patient level, and the analysis therefore treats calls as the sampling unit (see Limitations).

### 2.3. Outcome and Variables

The primary endpoint was field disposition, coded as hospitalization/transport to hospital (1) versus management at the scene or ambulatory care without hospital transfer (0). This endpoint reflects the operational decision made by the EMS crew and should be interpreted as an EMS transport/hospitalization decision, not as a patient-level clinical outcome. The dataset did not contain confirmed ACS diagnosis, in-hospital findings, mortality, re-contact after non-conveyance or adjudicated appropriateness of the decision. Candidate predictors, selected a priori on clinical and operational grounds, comprised: age (modeled both continuously and in categories <45, 45–59, 60–74 and ≥75 years), sex, clinical form of IHD (acute/unstable vs. chronic I25), the eight comorbidity/complication indicators listed above, the presence of any documented complication (DS3), dispatch urgency category (1–4), crew type (specialized vs. general/line) and EMS response time (modeled per additional 10 min). For the regression model, dispatch urgency was dichotomized into high urgency (categories 1–2) versus lower urgency (categories 3–4) because categories 1–2 correspond to high-priority, time-sensitive response in the local dispatch protocol and because category 1 and category 4 were sparse. The four original urgency categories are still shown descriptively in Section 3.2. This dichotomy was therefore operational and model-stability driven, and it should not be interpreted as a complete measure of on-scene clinical severity. Two operational time intervals—call-to-arrival and arrival-to-hospital-delivery—were summarized descriptively.

### 2.4. Statistical Analysis

Continuous variables were summarized as median (interquartile range, IQR) and compared between hospitalized/transported and left-at-scene groups with the Mann–Whitney U test, given their skewed distributions. Categorical variables were summarized as counts and percentages and compared with the Pearson chi-square test. Following the analytical approach established in comparable registry studies of coronary disease [7,8,9], independent associations with hospitalization/transport were estimated with an explanatory multivariable logistic regression model entering all pre-specified covariates simultaneously. The model was specified to quantify independent associations rather than to derive or validate a deployable prediction rule. Results are reported as adjusted odds ratios (aOR) with 95% confidence intervals (CI). Overall model significance was assessed with the log-likelihood-ratio test, and explained variation with McFadden’s pseudo-R2. Model performance was characterized in terms of discrimination (area under the receiver-operating-characteristic curve, AUC), overall accuracy (Brier score, together with a scaled Brier score relative to a prevalence-only model) and calibration (calibration intercept and slope, with a calibration plot of observed versus predicted probability across deciles of predicted risk). A 95% confidence interval for the AUC was obtained by percentile bootstrap (2000 resamples), and internal validity was assessed by bootstrap internal validation (500 resamples, Harrell’s optimism-correction procedure) to estimate the optimism-corrected AUC. A two-sided *p*-value < 0.05 was considered statistically significant. Adjusted odds ratios were visualized as a forest plot on a logarithmic scale. Analyses were performed in Python (version 3.11.5; Python Software Foundation, Wilmington, DE, USA) using the statsmodels, scikit-learn and pandas libraries. Reporting followed the STROBE statement. The flow of records from the full set of EMS dispatch entries to the final analytical samples, including each exclusion step and the corresponding numbers, is summarized in Figure 1.

## 3. Results

### 3.1. Analytical Sample and Overall Disposition

After duplicate removal and exclusion of calls without an unambiguous final disposition, the analytical sample comprised 9985 EMS calls for ischemic heart disease (IHD). Of 12,304 raw EMS dispatch records coded to ICD-10 I20–I25, 2080 duplicate call records were removed to leave 10,224 unique calls, and a further 239 calls with an ambiguous disposition were excluded; the full cleaning cascade is shown in Figure 1. Overall, 2676 calls (26.8%) resulted in hospitalization/transport to hospital, whereas 7309 calls (73.2%) were managed at the scene or resulted in ambulatory care. This distribution supports the use of disposition as an operationally relevant EMS endpoint, but it does not provide evidence on confirmed ACS, mortality or the clinical appropriateness of individual transport and non-transport decisions.

### 3.2. Demographic, Clinical, Comorbidity and Operational Characteristics

Baseline characteristics by call outcome are presented in Table 1. The median age of the study population was 68 years (IQR 59–77), and women accounted for 55.1% of calls. Compared with patients left at the scene, hospitalized/transported patients were younger (median 65 years, IQR 56–73, vs. 70 years, IQR 60–79; *p* < 0.001) and more frequently male (56.1% vs. 40.8%; *p* < 0.001). The age gradient was most pronounced among patients aged ≥75 years, who represented 22.3% of hospitalized/transported calls but 34.4% of calls left at the scene.

Chronic IHD (ICD-10 I25) accounted for most EMS calls (95.8%), while acute/unstable forms of IHD accounted for 4.2% of the sample. Nevertheless, the clinical form differed markedly by disposition: acute/unstable IHD represented 11.3% of hospitalized/transported calls but only 1.7% of calls left at the scene (*p* < 0.001). Urgency category was also associated with disposition (*p* < 0.001), whereas crew type did not differ significantly in the univariable comparison (*p* = 0.593).

The comorbidity and complication profile was dominated by cardiac conditions. Heart failure was the most common coded comorbidity/complication (30.8% overall) and was more frequent among hospitalized/transported patients than among those left at the scene (37.8% vs. 28.2%; *p* < 0.001). Other arrhythmias were also more frequent in the hospitalized/transported group (25.0% vs. 21.0%; *p* < 0.001), and cardiogenic shock, although uncommon overall (1.0%), was strongly concentrated among hospitalized/transported calls (3.3% vs. 0.2%; *p* < 0.001). In contrast, diabetes mellitus, COPD/obstructive disease, arterial hypertension, atrial fibrillation and pneumonia did not differ significantly between the outcome groups in unadjusted comparisons. Call-to-arrival response intervals were similar between the two dispositions; the arrival-to-hospital-delivery interval is defined only for calls resulting in hospitalization/transport and was therefore not compared between groups (Table 1).

### 3.3. Multivariable Predictors of Emergency Hospitalization/Transport

**Figure 2:**
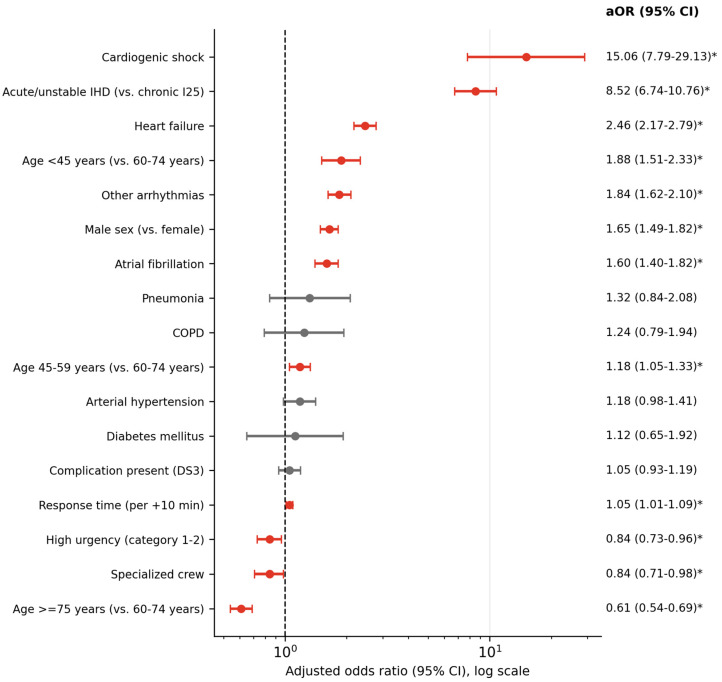
Adjusted odds ratios for hospitalization/transport among EMS calls for ischemic heart disease. Points represent aORs and horizontal bars represent 95% confidence intervals. The vertical dashed line denotes OR = 1; estimates to the right indicate higher adjusted odds and estimates to the left indicate lower adjusted odds. * *p* < 0.05.

The multivariable logistic regression model was statistically significant (likelihood-ratio test *p* < 0.001; McFadden pseudo-R2 = 0.093). Adjusted odds ratios (aORs) are shown graphically in Figure 2 and reported numerically in Table 2. As expected, acute severity variables showed the largest associations with hospitalization/transport; the additional analytic value of the model was to quantify whether routinely coded comorbidity variables retained independent associations after adjustment for acuity, demographic and operational factors.

Cardiogenic shock was the strongest independent predictor of hospitalization/transport (aOR 15.06; 95% CI 7.79–29.13; *p* < 0.001), followed by acute/unstable IHD compared with chronic I25-coded disease (aOR 8.52; 95% CI 6.74–10.76; *p* < 0.001). Among cardiac comorbidity indicators, heart failure (aOR 2.46; 95% CI 2.17–2.79), other arrhythmias (aOR 1.84; 95% CI 1.62–2.10) and atrial fibrillation (aOR 1.60; 95% CI 1.40–1.82) remained independently associated with higher odds of hospitalization/transport after adjustment for demographic, clinical and operational variables.

Demographic and operational covariates also contributed to the final model. Male sex was associated with increased odds of hospitalization/transport (aOR 1.65; 95% CI 1.49–1.82), as was age < 45 years compared with the 60–74-year reference group (aOR 1.88; 95% CI 1.51–2.33). By contrast, age ≥ 75 years was associated with lower adjusted odds (aOR 0.61; 95% CI 0.54–0.69). Each additional 10 min of EMS response time was associated with a modest increase in the adjusted odds of hospitalization/transport (aOR 1.05; 95% CI 1.01–1.09).

### 3.4. Model Performance and Internal Validation

**Figure 3:**
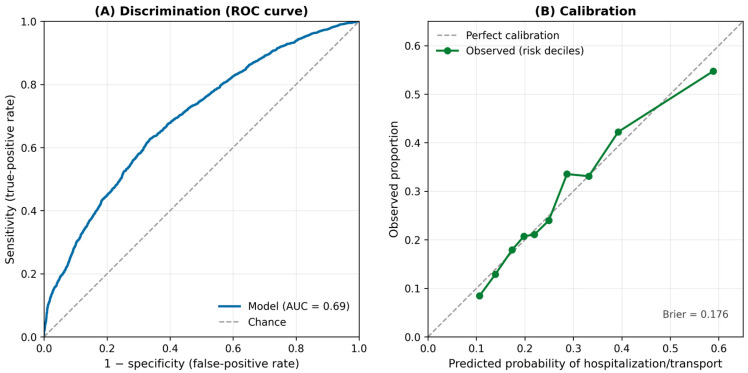
Performance of the explanatory multivariable logistic regression model for hospitalization/transport (development sample, N = 9860). (**A**) Receiver-operating-characteristic (ROC) curve; the area under the curve (AUC) was 0.69 (95% CI 0.68–0.70), indicating moderate discrimination. (**B**) Calibration of predicted versus observed probability of hospitalization/transport across deciles of predicted risk; points lie close to the line of perfect calibration and the Brier score was 0.18. Bootstrap internal validation (500 resamples) gave an optimism-corrected AUC of 0.69.

Because the model was specified to explain rather than to predict the at-scene disposition, its performance is reported to describe model fit rather than to support a deployable prediction tool. Discrimination was moderate: the area under the receiver-operating-characteristic curve (AUC) was 0.69 (95% CI 0.68–0.70; Figure 3A). Overall accuracy of the predicted probabilities was modest, with a Brier score of 0.18 and a scaled Brier score of 0.10 relative to a prevalence-only model. Calibration across deciles of predicted risk was close to the identity line over the observed probability range (Figure 3B), with an apparent calibration slope of 1.00 and intercept of 0.00 on the development data. Bootstrap internal validation (500 resamples, Harrell’s optimism-correction procedure) indicated minimal overfitting, with a mean optimism of 0.004 and an optimism-corrected AUC of 0.69. The AUC should be interpreted cautiously: it is below the approximate 0.74–0.79 reported for dedicated prehospital chest-pain triage models [11,12] and indicates that routinely coded dispatch variables explain only part of the transport decision. The predictors identified here are therefore best treated as candidate variables for future model development, not as a validated risk-stratification instrument.

### 3.5. Graphical Summary of Clinical Disposition and Comorbidity Burden

**Figure 4:**
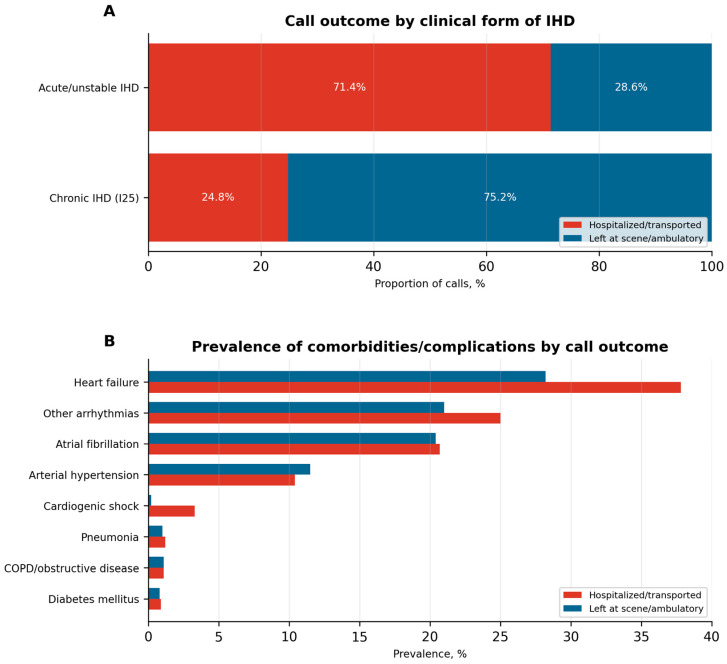
Clinical form, EMS disposition and comorbidity burden. (**A**) Distribution of call outcomes by clinical form of ischemic heart disease. (**B**) Prevalence of selected comorbidities and complications by final call outcome. Percentages are calculated within each outcome or clinical subgroup.

The graphical summaries complement the tabular findings by showing how disposition differs by clinical form and comorbidity profile. Acute/unstable IHD calls had a substantially higher hospitalization/transport proportion than chronic IHD calls (71.4% vs. 24.8%; Figure 4A). The hospitalized/transported group also had a higher prevalence of heart failure, other arrhythmias and cardiogenic shock, whereas diabetes mellitus, COPD/obstructive disease and pneumonia were rare in both outcome groups (Figure 4B).

### 3.6. Age Distribution and Annual Dynamics

**Figure 5:**
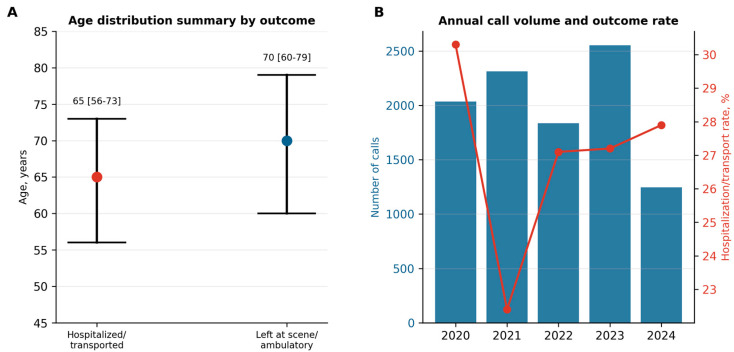
Age and annual dynamics of EMS calls for ischemic heart disease. (**A**) Median age and interquartile range by final call outcome. (**B**) Annual call volume and hospitalization/transport rate during the study period. Data for 2020 begin on 19 February and data for 2024 cover 1 January to 30 June only, so the lower call counts in those two years partly reflect incomplete calendar coverage rather than a true change in volume.

Age and annual patterns are summarized in Figure 5. The median-IQR plot confirms the younger profile of hospitalized/transported patients compared with those left at the scene (Figure 5A), consistent with the values reported in Table 1. Annual call volume varied across the observation period, while the hospitalization/transport rate declined in 2021 and then partially recovered in subsequent years (Figure 5B).

Overall, hospitalization/transport among EMS calls for IHD was associated with both the acute clinical form of IHD and specific cardiac comorbidity/complication profiles. Cardiogenic shock and acute/unstable IHD were the dominant, clinically expected acuity-related predictors, while heart failure, other arrhythmias and atrial fibrillation remained independent comorbidity-related predictors after adjustment. Diabetes mellitus and COPD were rare in the coded EMS fields and did not show statistically significant adjusted associations in this dataset, suggesting limited capture of non-cardiac comorbidity in the available DS2/DS3 coding structure.

## 4. Discussion

In this five-year call-level analysis of 9985 EMS activations for IHD in Astana, the largest associations with hospitalization/transport were clinically expected: cardiogenic shock and acute/unstable IHD. The main contribution of the study is therefore more specific: it quantifies how routinely coded cardiac comorbidity and complication fields, recorded during real EMS work, remain independently associated with the transport decision after adjustment for acuity, demographic and operational variables. This endpoint is the crew-side disposition decision, not a clinical outcome or an adjudication of whether transport was appropriate. The findings should consequently be interpreted as explanatory evidence about EMS decision patterns rather than as direct evidence of patient prognosis or a ready decision-support rule.

### 4.1. Acuity, Comorbidity and the Wider Evidence

That hemodynamic instability and acute presentation dominated the model is concordant with clinical logic and the broader coronary literature: cardiogenic shock is the archetypal high-acuity complication, and contemporary staging systems and registries consistently report high in-hospital mortality, justifying escalation of care [25,26,27]. Position statements on AMI complicated by cardiogenic shock likewise frame immediate transfer to definitive care as the default [28]. The less self-evident finding concerns stable cardiac comorbidity. The independent association of heart failure with hospitalization/transport aligns with evidence showing that heart failure is a principal driver of emergency presentation, admission and readmission, and that regression- and machine learning-based tools can anticipate heart-failure ED visits and hospitalizations [19,20,22]. The independent contributions of atrial fibrillation and other arrhythmias are also plausible because admission rates for atrial fibrillation increase in the presence of concomitant heart failure and multimorbidity [23,24]. More generally, multimorbidity is strongly associated with emergency admission and short-term mortality, and comorbidity-based scores such as the DICER-score have been developed for emergency-department demand stratification [17,18]. Our data extend this literature to the prehospital transport decision in a Central Asian urban EMS system, while recognizing that the associations reflect EMS disposition patterns rather than independently verified outcomes.

### 4.2. Response Time, Demography and the Crew-Side Decision

The association between longer response time and higher hospitalization/transport odds parallels the Beijing driving-time study, in which longer travel to PCI-capable hospitals raised case-fatality odds in graded fashion [9]; in both settings, time behaves partly as a proxy for case severity and geographic remoteness rather than as a simple causal exposure. This interpretation is also consistent with local GIS-based evidence from Astana showing that urban environmental and spatiotemporal factors shape EMS response patterns [32], while telemedicine- and system-delay studies have similarly emphasized the importance of operational delay [33]. The demographic pattern we observed merits particular comment. Studies of patient-driven prehospital delay generally find that older age and female sex are associated with later presentation and poorer access [7,29,30], whereas our crew-side outcome showed younger and male patients more likely to be conveyed and the oldest patients conveyed less often. The divergence is informative rather than contradictory: delay studies capture help-seeking behavior, while our outcome captures the hospitalization/transport decision made once the crew is on scene. Lower hospitalization/transport odds among patients aged ≥75 years may reflect a higher prevalence of chronic stable disease managed at home, ceilings-of-care considerations, frailty-related decisions or patient preference, and should be interpreted cautiously rather than read as simple under-triage—particularly because multimorbidity confers proportionally greater risk in younger patients [18,24]. Sex differences in symptom presentation and in delayed hospitalization reported for ACS and NSTEMI reinforce that demographic effects on prehospital outcomes are context-dependent and should be modeled explicitly [29,30].

Two operational associations ran counter to naive expectation and warrant explicit interpretation. First, high dispatch urgency (categories 1–2) was associated with slightly lower adjusted odds of hospitalization/transport (aOR 0.84). The dichotomy was chosen a priori for operational and statistical reasons: categories 1–2 represent the higher-priority response levels in the local dispatch protocol, while category 1 and category 4 were sparse. However, the combined high-urgency group was dominated by category 2, which accounted for 81.0% of all calls, and it was contrasted with a small, heterogeneous category 3–4 group. This imbalance can compress the contrast and plausibly pull the adjusted estimate below 1. Dispatch urgency is also assigned before direct clinical assessment, from limited caller information, and therefore differs conceptually from on-scene severity. The inverse coefficient should therefore not be read as evidence that higher urgency protects against transport; it more likely reflects classification structure, case-mix and residual confounding. Second, attendance by a specialized crew was associated with marginally lower adjusted odds of hospitalization/transport (aOR 0.84). This also should not be interpreted causally. Specialized crews are deployed non-randomly and may stabilize selected patients on scene, while their case mix is shaped by dispatch protocols. Both operational estimates are therefore endogenous to the dispatch and deployment process; only future analyses modeling urgency as all four categories, or using quasi-experimental designs, could determine whether these variables have an independent causal effect on transport.

### 4.3. Methodological Context and Implications

Methodologically, our study sits within an active stream of work building prehospital risk-stratification and dispatch models. Logistic regression and machine learning models for chest-pain triage and dispatch have achieved AUCs of roughly 0.74–0.79 using variables routinely available to EMS [11,12], and models using the prehospital electrocardiogram or non-conveyance follow-up data have performed similarly or modestly better while often reducing interpretability [10,13,14]. Against that benchmark, the present AUC of 0.69 is not sufficient for clinical deployment. The practical implication is therefore limited and developmental: heart failure, arrhythmias and cardiogenic shock should be evaluated as candidate features in future models that also include vital signs, ECG findings, symptom severity, prehospital treatment and linked hospital outcomes. Until such models are prospectively validated, comorbidity-based decision tools should not be implemented for individual transport decisions. At the service-planning level, however, aggregated comorbidity patterns may help structure audit questions, identify documentation gaps and design safer evaluations of dispatch prioritization and crew allocation, while remaining mindful that both over-triage and under-triage carry measurable risk [15,16,34,35,36].

### 4.4. Strengths and Limitations

The principal strengths of this study are its large, consecutive, population-based sample drawn from a centralized municipal dispatch system, its five-year span, its STROBE-compliant reporting [37], and the use of a pre-specified multivariable model that mirrors the analytical standard of comparable coronary registries [7,8,9]. Capturing the real at-scene hospitalization/transport decision, rather than a downstream hospital outcome, is a further strength with direct operational relevance, and the setting addresses a genuine evidence gap for Central Asian EMS systems [5,6].

Several limitations temper these strengths and directly address the interpretation of the findings. First, the endpoint was field disposition—hospitalization/transport versus management at the scene—not confirmed ACS, in-hospital diagnosis, mortality, readmission or an adjudicated measure of decision appropriateness. We therefore cannot determine whether transported patients truly required admission or whether non-conveyed patients were safely managed; non-conveyance safety can only be assessed through linkage to re-contact, hospital and mortality outcomes [14,15,16]. Second, the analysis relies on working diagnoses and comorbidity indicators recorded by ambulance crews under time pressure. Misclassification and under-documentation are likely, as illustrated by the very low recorded prevalence of diabetes mellitus (0.8%) and COPD/obstructive disease (1.1%), which almost certainly reflect incomplete field coding rather than true comorbidity prevalence among patients with IHD. Such non-differential under-capture would tend to attenuate associations toward the null, so near-unity estimates for diabetes and COPD should not be interpreted as evidence of no clinical relevance. Third, important clinical variables were unavailable, including vital signs, 12-lead ECG findings, symptom characteristics and severity, pain scores, point-of-care troponin or other laboratory data, frailty measures, patient preference and prehospital treatments. Their absence is the most plausible explanation for the moderate discrimination observed (AUC 0.69) and sets an inherent ceiling for models based only on routine dispatch fields. The model should therefore be regarded as an explanatory association model, not as a deployable triage or risk-stratification instrument. Fourth, response time, dispatch urgency and crew type are partly endogenous to severity and dispatch protocols, so their adjusted odds ratios should be interpreted as conditional associations rather than causal effects. Fifth, the unit of analysis was the EMS call rather than the unique patient because the dispatch dataset lacked a stable cross-call patient identifier. Repeat calls could therefore not be clustered or collapsed, which may underestimate standard errors and narrow confidence intervals. Finally, the data derive from a single capital-city EMS system. Dispatch algorithms, crew scope of practice, hospital-network density, admission thresholds and non-conveyance protocols differ across systems; therefore, the specific adjusted odds ratios may not transfer directly to rural Kazakhstan, other Central Asian services or other health systems. Multicenter studies with harmonized definitions, patient-level linkage and prospective validation are needed to test generalizability.

### 4.5. Future Directions

Future work should first link prehospital records to hospital and mortality registries to evaluate the appropriateness and safety of transport and non-transport decisions. Subsequent models should incorporate vital signs, ECG findings, symptom severity, prehospital treatment and point-of-care biomarkers, and should be externally and prospectively validated before any use in individual triage. Only after that validation should comorbidity-aware risk stratification be considered for decision support; until then, these variables are best used for hypothesis generation, documentation improvement and service-level planning. Comparative studies across urban and rural EMS systems in Central Asia would help establish the generalizability of the predictors identified here.

## 5. Conclusions

In this cohort of 9985 EMS calls for ischemic heart disease in Astana, approximately one in four calls resulted in hospitalization/transport. The largest associations were the clinically expected acuity markers—cardiogenic shock and acute/unstable IHD—while routinely coded cardiac comorbidities, especially heart failure, atrial fibrillation and other arrhythmias, were independently associated with the EMS transport decision after adjustment. Male sex and younger age were associated with higher transport odds, whereas the oldest age group, specialized crews and higher dispatch urgency were associated with lower odds. These findings describe call-level EMS disposition patterns and should not be interpreted as patient outcomes or as evidence that any individual transport decision was clinically correct. Because model discrimination was only moderate and key clinical variables were unavailable, the results do not support the immediate implementation of a comorbidity-based triage tool. Instead, they identify candidate variables for future risk-stratification models that must include richer clinical data, linkage to patient outcomes and prospective validation before clinical use.

## Figures and Tables

**Table 1:** Demographic, clinical, comorbidity and operational characteristics by EMS call outcome.

Characteristic	Total	Hospitalized/ Transported	Left at Scene/ Ambulatory	*p*-Value	Test
n (% of sample)	9985	2676 (26.8%)	7309 (73.2%)		
Age, median (IQR), years	68 (59–77)	65 (56–73)	70 (60–79)	<0.001	Mann–Whitney U
Sex, n (%)				<0.001	Pearson chi-square
Male	4484 (44.9%)	1502 (56.1%)	2982 (40.8%)		
Female	5501 (55.1%)	1174 (43.9%)	4327 (59.2%)		
Age group, n (%)				<0.001	Pearson chi-square
<45	436 (4.4%)	193 (7.2%)	243 (3.3%)		
45–59	2205 (22.1%)	719 (26.9%)	1486 (20.3%)		
60–74	4231 (42.4%)	1166 (43.6%)	3065 (41.9%)		
≥75	3113 (31.2%)	598 (22.3%)	2515 (34.4%)		
Urgency category, n (%)				<0.001	Pearson chi-square
Category 1	186 (1.9%)	65 (2.4%)	121 (1.7%)		
Category 2	8091 (81.0%)	2106 (78.7%)	5985 (81.9%)		
Category 3	1444 (14.5%)	452 (16.9%)	992 (13.6%)		
Category 4	264 (2.6%)	53 (2.0%)	211 (2.9%)		
Main diagnosis, n (%)				<0.001	Pearson chi-square
Acute/unstable IHD	423 (4.2%)	302 (11.3%)	121 (1.7%)		
Chronic IHD (I25)	9562 (95.8%)	2374 (88.7%)	7188 (98.3%)		
Crew type, n (%)				0.593	Pearson chi-square
Specialized	1074 (10.8%)	280 (10.5%)	794 (10.9%)		
General/line	8911 (89.2%)	2396 (89.5%)	6515 (89.1%)		
Comorbidities/complications, n (%)					
Diabetes mellitus (E10–E14)	83 (0.8%)	23 (0.9%)	60 (0.8%)	0.949	Pearson chi-square
COPD/obstructive disease (J40–J47)	109 (1.1%)	29 (1.1%)	80 (1.1%)	1.000	Pearson chi-square
Arterial hypertension (I10–I15)	1118 (11.2%)	279 (10.4%)	839 (11.5%)	0.149	Pearson chi-square
Heart failure (I50)	3071 (30.8%)	1011 (37.8%)	2060 (28.2%)	<0.001	Pearson chi-square
Atrial fibrillation (I48)	2043 (20.5%)	555 (20.7%)	1488 (20.4%)	0.696	Pearson chi-square
Other arrhythmias (I47, I49)	2204 (22.1%)	669 (25.0%)	1535 (21.0%)	<0.001	Pearson chi-square
Cardiogenic shock (R57.0)	99 (1.0%)	88 (3.3%)	11 (0.2%)	<0.001	Pearson chi-square
Pneumonia (J12–J18)	106 (1.1%)	32 (1.2%)	74 (1.0%)	0.495	Pearson chi-square
Time intervals, median (IQR), min					
Call to crew arrival	14 (11–16)	14 (11–16)	14 (11–16)	0.589	Mann–Whitney U
Arrival to hospital delivery (transported calls only)	62 (52–74)	62 (52–74)	—	—	—

Values are n (%) unless otherwise indicated. Continuous variables are summarized as median (interquartile range, IQR) and compared using the Mann–Whitney U test. The arrival-to-hospital-delivery interval is defined only for calls resulting in hospitalization/transport and is reported for those calls only; it is not applicable to calls managed at the scene, so no between-group comparison is presented for this interval. Categorical variables are compared using the Pearson chi-square test. Exact *p*-values are shown when *p* ≥ 0.001; values below 0.001 are reported as *p* < 0.001. Calls with death before arrival, handover or another ambiguous disposition were excluded from outcome comparisons. COPD: chronic obstructive pulmonary disease; EMS: emergency medical service; IHD: ischemic heart disease.

**Table 2:** Multivariable logistic regression model for predictors of hospitalization/transport.

Predictor	aOR	95% CI	*p*-Value
Cardiogenic shock	15.06	7.79–29.13	<0.001
Acute/unstable IHD (vs. chronic I25)	8.52	6.74–10.76	<0.001
Heart failure	2.46	2.17–2.79	<0.001
Age < 45 years (vs. 60–74 years)	1.88	1.51–2.33	<0.001
Other arrhythmias	1.84	1.62–2.10	<0.001
Male sex (vs. female)	1.65	1.49–1.82	<0.001
Atrial fibrillation	1.60	1.40–1.82	<0.001
Pneumonia	1.32	0.84–2.08	0.228
COPD	1.24	0.79–1.94	0.352
Age 45–59 years (vs. 60–74 years)	1.18	1.05–1.33	0.006
Arterial hypertension	1.18	0.98–1.41	0.080
Diabetes mellitus	1.12	0.65–1.92	0.681
Complication present (DS3)	1.05	0.93–1.19	0.407
Response time (per +10 min)	1.05	1.01–1.09	0.012
High urgency (category 1–2)	0.84	0.73–0.96	0.012
Specialized crew	0.84	0.71–0.98	0.029
Age ≥ 75 years (vs. 60–74 years)	0.61	0.54–0.69	<0.001

Dependent variable: hospitalization/transport to hospital = 1. Reference categories: female sex, age 60–74 years, chronic IHD (I25), non-high urgency, general/line crew and absence of the respective comorbidity/complication. Model N = 9860. aOR: adjusted odds ratio; CI: confidence interval; COPD: chronic obstructive pulmonary disease; IHD: ischemic heart disease.

## Data Availability

The data presented in this study are available from the corresponding author upon reasonable request. Due to institutional data-use agreements, ethics requirements, and privacy considerations, the underlying retrospective emergency medical service records cannot be publicly shared, as there remains a potential risk of indirect participant re-identification.

## Abbreviations

The following abbreviations are used in this manuscript:

ACS	Acute coronary syndrome
AMI	Acute myocardial infarction
aOR	Adjusted odds ratio
AUC	Area under the curve
CI	Confidence interval
COPD	Chronic obstructive pulmonary disease
DS2	Secondary diagnosis coding field
DS3	Documented complication coding field
ED	Emergency department
EMS	Emergency medical service
ICD-10	International Classification of Diseases, 10th Revision
IHD	Ischemic heart disease
IQR	Interquartile range
NSTEMI	Non-ST-elevation myocardial infarction
OR	Odds ratio
PCI	Percutaneous coronary intervention
ROC	Receiver-operating-characteristic
STEMI	ST-elevation myocardial infarction
STROBE	Strengthening the Reporting of Observational Studies in Epidemiology
